# Priority domains, aims, and testable hypotheses for implementation research: Protocol for a scoping review and evidence map

**DOI:** 10.1186/s13643-020-01535-y

**Published:** 2020-12-03

**Authors:** Bryan R. Garner, Sheila V. Patel, M. Alexis Kirk

**Affiliations:** 1 RTI International, P. O. Box 12194, Research Triangle Park, NC 27709-2194 USA; 2 Centerstone Research Institute, 44 Vantage Way, Suite 400, Nashville, TN 37228 USA

**Keywords:** Implementation science, Implementation research, Priority setting

## Abstract

**Background:**

The challenge of implementing evidence-based innovations within practice settings is a significant public health issue that the field of implementation research (IR) is focused on addressing. Significant amounts of funding, time, and effort have been invested in IR to date, yet there remains significant room for advancement, especially regarding IR’s development of scientific theories as defined by the National Academy of Sciences (i.e., a comprehensive explanation of the relationship between variables that is supported by a vast body of evidence). Research priority setting (i.e., promoting consensus about areas where research effort will have wide benefits to society) is a key approach to helping accelerate research advancements. Thus, building upon existing IR, general principles of data reduction, and a general framework for moderated mediation, this article identifies four priority domains, three priority aims, and four testable hypotheses for IR, which we organize in the priority aims and testable hypotheses (PATH) diagram.

**Methods:**

The objective of this scoping review is to map the extent to which IR has examined the identified PATH priorities to date. Our sample will include IR published in leading implementation-focused journals (i.e., *Implementation Science*, *Implementation Science Communications*, and *Implementation Research and Practice*) between their inception and December 2020. The protocol for the current scoping review and evidence map has been developed in accordance with the approach developed by Arksey and O’Malley and advanced by Levac, Colquhoun, and O’Brien. Because scoping reviews seek to provide an overview of the identified evidence base rather than synthesize findings from across studies, we plan to use our data-charting form to provide a descriptive overview of implementation research to date and summarize the research via one or more summary tables. We will use the PATH diagram to organize a map of the evidence to date.

**Discussion:**

This scoping review and evidence map is intended to help accelerate IR focused on suggested priority aims and testable hypotheses, which in turn will accelerate IR’s development of National Academy of Sciences-defined scientific theories and, subsequently, improvements in public health.

**Systematic review registration:**

Open Science Framework https://osf.io/3vhuj/

## Background

The persistence of unacceptably low rates of translating research findings into practice has led to increasing attention to implementation research (IR) as a means to significantly accelerate improvements in public health [1, 2]. Over a decade ago, Eccles and Mittman defined IR as “the scientific study of methods to promote the systematic uptake of research findings and other evidence-based practices into routine practice, and, hence, to improve the quality and effectiveness of health services and care” [3]. Similarly, the National Institutes of Health has consistently defined IR as “the scientific study of the use of strategies to adopt and integrate evidence-based health interventions into clinical and community settings in order to improve patient outcomes and benefit population health” [4, 5]. Considering the significant amounts of funding, time, and effort invested in IR, it would be ideal if the field of IR had developed one or more scientific theories as defined by the National Academy of Sciences (i.e., a comprehensive explanation of the relationship between variables that is supported by a vast body of evidence) [6]. Although the field of IR has developed various theories, models, and frameworks to support IR, these theories, models, and frameworks have some limitations [7, 8]. First, they tend to be narrow in scope, focusing on one area of implementation research (e.g., evaluation, implementation determinants, implementation processes), instead of comprehensive explanations of phenomena. Second, although there are many models and frameworks, there are few theories (comprehensive explanations of relationships between variables) and, to our knowledge, no IR theories are supported by vast bodies of evidence the way prominent theories in other fields are (e.g., Theory of Planned Behavior, which has been widely applied across fields to predict human social behavior and has a vast body of evidence, including meta-analyses assessing the predictive validity of its theoretical propositions) [9, 10]. Given the limitations of IR theories, efforts to accelerate the development of theories that meet National Academy of Sciences standards are warranted.

Research priority setting (i.e., promoting consensus about areas where research effort will have wide benefits to society) is one approach to accelerating research advancements [11]. The Priority Aims and Testable Hypotheses for IR (PATH4IR) Project seeks to accelerate IR on several of IR’s priority domains, aims, and testable hypotheses via estimating the extent to which IR to date has examined these priority areas. Helping accelerate IR on these priorities should accelerate IR’s development of National Academy of Sciences-defined scientific theories, which in turn will help accelerate improvements in public health. Below, we identify, describe, and justify four priority domains, three priority aims, and four priority testable hypotheses for IR, which are the focus of the PATH4IR Project and its scoping review.

### Four priority domains for implementation research

A plethora of IR theories, models, and frameworks have identified numerous IR domains [7, 12]. Table 1 lists the domains of three IR models/frameworks that have guided much IR to date. Building on these IR models/frameworks [13–15], other IR [16], principles of data reduction [17], and a general framework for moderated mediation [18], the PATH4IR Project identified four priority domains for IR. Each priority domain is defined below and in Table 2.

**Table 1:** Domains included in several existing implementation research models/frameworks

Implementation research model/framework	List of domains
Proctor et al. (2009)—a conceptual model of implementation research [13]	Intervention strategies, implementation strategies, outcomes
Damschroder et al. (2009)—the consolidated framework for implementation research [14]	Intervention characteristics, outer setting, inner setting, characteristics of the individuals involved, process of implementation
Aarons et al. (2011)—conceptual model of evidence-based practice implementation in public service sectors [15]	Outer context, inner context, innovation characteristics and intervention developers, innovation/system fit, innovation/organization fit, interconnections

**Table 2:** The priority domains for implementation research

Priority domain (acronym)	Brief description	Justification
Implementation strategies (IS)	Strategies used to put into practice a program of known dimensions (e.g., EBP)	[13, 19]
Evidence-based measures of implementation (EBMI)	A measure shown to be predictive of improvement in one or more key HHROs (e.g., client outcomes)	[20]
Health and health-related outcomes (HHRO)	End-points regarding evidence-based process of care, client/patient outcomes, or population outcomes	[13, 21]
Context-related moderators/mediators (CRMM)	Measures of the outer setting/context or inner setting/context that are hypothesized to moderate and/or mediate relationships between the other domains (i.e., IS, HHRO, EBMI)	[14, 15]

*IS* implementation strategies, *EBMI* evidence-based measure of implementation, *HHRO* health and health-related outcomes, *CRRM* context-related moderators/mediators, *EBP* evidence-based practice

#### Implementation strategies

Implementation strategies are defined as the strategies used to put into practice a program of known dimensions (e.g., an evidence-based practice [EBP]) [13, 19]. Given how IR has been defined and that implementation strategies are the quintessential independent variable in IR [3–5], we consider the implementation strategy (IS) domain a priority for IR.

#### Evidence-based measures of implementation

If implementation strategies are the quintessential independent variable of IR, implementation outcomes have become the quintessential dependent variable. However, consistent with the important distinction demonstrated between a practice and an EBP [22], an important distinction has been demonstrated between an implementation outcome and an evidence-based measure of implementation (EBMI) [20]. An implementation outcome is defined as “the effects of deliberate and purposive actions to implement new treatments, practices, and services,” [16] whereas an EBMI is defined as “an implementation outcome measure that is predictive of improvements in key client outcomes” (i.e., health and health-related outcomes [HHROs], such as client functioning, health-related quality of life, or morbidity/mortality) [20]. This means that while all EBMIs are implementation outcomes, not all implementation outcomes are EBMIs. IR has historically prioritized implementation outcomes, but as noted by Proctor and colleagues, implementation outcomes should not be treated as dependent variables until we have advanced them as consistent, valid, and efficient measures of implementation [16]. Otherwise, we rely on the assumption that implementation outcomes are predictive of HHROs, without empirically demonstrating this to be true. To our knowledge, the PATH4IR Project is the first to explicitly identify EBMIs as a priority domain for IR.

#### Health and health-related outcomes

Health outcomes (e.g., client/patient functioning) and health-related outcomes (e.g., health-related quality of life, quality adjusted life years) are the outcomes that IR seeks to ultimately improve. Despite this, not all outcome-focused IR models/frameworks explicitly include the HHRO domain [14, 15]. Instead, many focus on implementation outcomes, leaving out HHROs entirely. We identify HHROs as a priority domain for IR for two reasons. First, as noted above, until EBMIs are established, measuring only implementation outcomes relies on the assumption that implementation outcomes are predictive of HHROs. Second, as noted by Foy et al., “If studies evaluating the effects of implementation interventions are to be of relevance to policy and practice, they should have end-points related to evidence-based processes of care.” [21].

#### Context-related moderators/mediators

Moderation occurs when the effect of an independent measure on a dependent variable depends on the level of another measure, and mediation occurs when the effect of an independent variable on a dependent measure is transmitted through a third variable [23]. Given that existing IR models/frameworks have highlighted the importance of context [14, 15, 24] and that Edwards and Lambert’s general framework for moderated mediation [18] guided identification of the priority domains for this project, context-related moderators/mediators (CRMMs) were identified as a priority domain for IR. Conceptualizing context as potential moderators/mediators (instead of just discrete factors that “influence” implementation) moves the field of IR towards National Academy of Sciences-consistent theory as it starts to clarify relationships between constructs.

### Three priority aims for implementation research

**Fig. 1:**
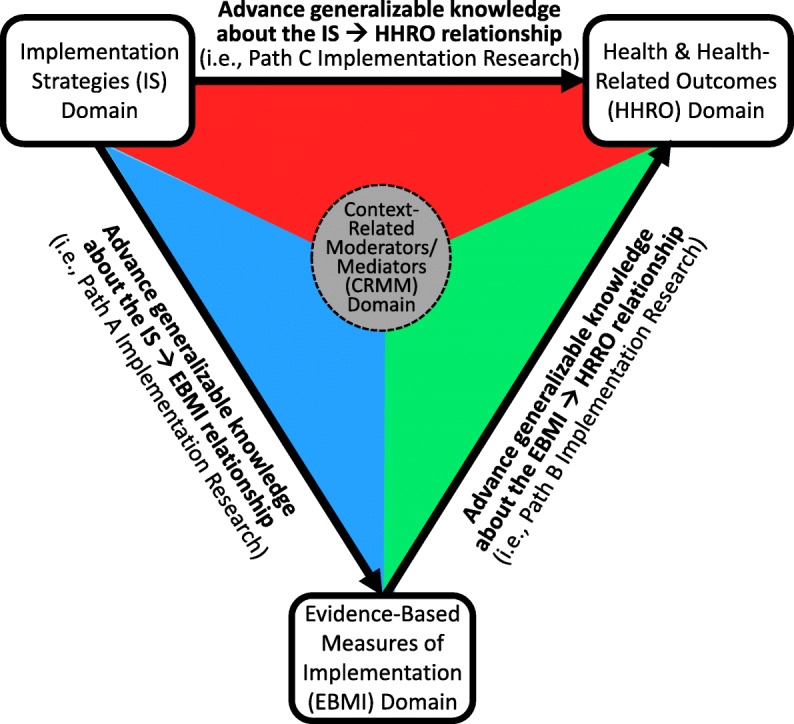
Priority aims for implementation research

There are numerous aims (i.e., research questions) that IR could address, and there is value in establishing consensus regarding the types of aims IR should prioritize. Relative to IR’s domains, IR’s aims have received less explicit attention. The work of Curran et al. [25] is one exception. Specifically, for their type 3 effectiveness-implementation research categorization, Curran et al. recommended that the primary aim of this research category was to “determine utility of an implementation intervention/strategy,” and the secondary aim was to “assess clinical outcomes associated with implementation trial” [25]. Curran et al. also recommended implementation outcomes (e.g., adoption, fidelity) as dependent measures for the primary aim, with client outcomes (e.g., patient symptoms patient functioning) as dependent measures for the secondary aim [25]. However, priority aims have not generally been explicitly addressed by most other IR models/frameworks [13–15]. Given that developing or contributing to generalizable knowledge is central to how research is defined [26], it is important that IR prioritize aims that seek to develop or contribute to generalizable knowledge for its priority relationships. Thus, building from the four priority domains described above, we identified the following three priority aims for IR: (1) the IS to HHRO relationship (i.e., IS ➔ HHRO), (2) the IS to EBMI relationship (i.e., IS ➔ EBMI), and (3) the EBMI to HHRO (i.e., EBMI ➔ HHRO). Consistent with mediational analysis literature [27–30] we have termed IR focused on the IS ➔ HHRO relationship as *Path C IR* (the red triangle of Fig. 1), IR focused on the IS ➔ EBMI relationship as *Path A IR* (the blue triangle of Fig. 1), and IR focused on the EBMI ➔ HHRO relationship as *Path B IR* (the green triangle of Fig. 1). Each priority aim is defined below and in Table 3.

**Table 3:** The priority aims for implementation research

Priority aim	Type
Advance generalizable knowledge regarding the IS ➔ HHRO relationship	Path C implementation research
Advance generalizable knowledge regarding the IS ➔ EBMI relationship	Path A implementation research
Advance generalizable knowledge regarding the EBMI ➔ HHRO relationship	Path B implementation research

*IS* implementation strategies, *HHRO* health and health-related outcomes, *EBMI* evidence-based measures of implementation

#### Advance generalizable knowledge regarding the IS ➔ HHRO relationship

Advancing generalizable knowledge about the relationship between an IS and a HHRO is termed *Path C IR*. Given IR’s emphasis on strategies to increase the uptake of EBPs to improve patient and population health [3–5] and the importance of measuring outcomes that have relevance to policy and practice [21], *Path C IR* was identified as a priority aim for IR. An example of *Path C IR* is a 29-site cluster-randomized implementation experiment Garner et al. conducted between 2008 and 2012 that focused on testing the impact of a pay-for-performance implementation strategy to improve the implementation and effectiveness of the Adolescent Community Reinforcement Approach (A-CRA), which is an EBP for adolescents with substance use disorders [31]. The dependent variable of interest was an HHRO—adolescent substance use recovery status at 6-month follow-up.

#### Advance generalizable knowledge regarding the IS ➔ EBMI relationship

Advancing generalizable knowledge about the relationship between an IS and an EBMI is termed *Path A IR*. Given that an EBMI is a measure of EBP implementation found to be predictive of key client outcomes [20], *Path A IR* was identified as a priority aim for IR. Relative to IR that has tested the impact of an IS on implementation outcomes that do not have evidence of being a meaningful predictor of key client outcomes, IR testing the impact of an IS on EBMIs appears be limited. Having established an EBMI for A-CRA as part of an effectiveness study [32, 33], Garner et al. [31] also provide an example of *Path A IR*. Examining the impact of pay-for-performance on an EBMI called Target A-CRA (i.e., 10+ of the core the A-CRA components delivered within no less than seven sessions), which prior research found to be significantly associated with greater reductions in adolescents’ days of abstinence at follow-up [32], Garner et al. found that relative to adolescents in the implementation-as-usual condition, adolescents in the pay-for-performance condition had a significantly higher likelihood of receiving Target A-CRA [31].

#### Advance generalizable knowledge regarding the EBMI ➔ HHRO relationship

Advancing generalizable knowledge about the relationship between an EBMI and HHRO is termed *Path B IR*. Research by Nosek et al. [34], which increased concern regarding the reproducibility of psychological science, underscores why *Path B IR* is a priority. That is, it is important that significant relationships (e.g., EBMI ➔ HHRO) supported as part of effectiveness research be examined for replicability within IR. As part of their IR experiment, Garner et al. [31] provide an example of *Path B IR* by replicating a significant association between target A-CRA (i.e., the previously established evidence-based measure of implementation) and adolescent abstinence from substance use at follow-up (i.e., the HHRO) [31].

### Four priority testable hypotheses for implementation research

**Fig. 2:**
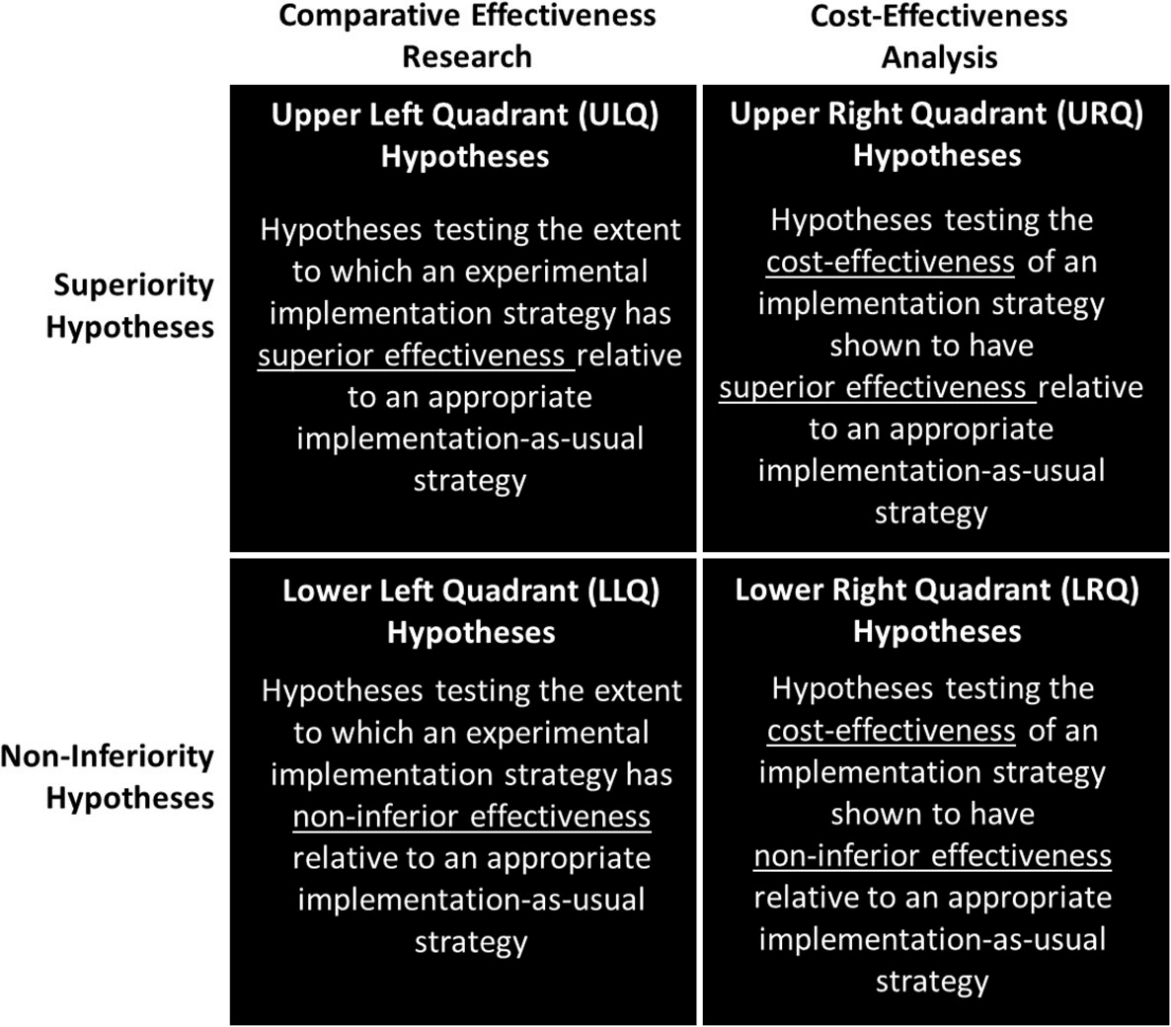
Priority hypotheses for implementation research

While the possible testable hypotheses for IR are numerous, there is value in establishing consensus regarding the types of testable hypotheses IR should prioritize. Toward helping generate National Academy of Sciences-defined *scientific* IR, prioritizing one or more of the four testable hypotheses shown in Fig. 2 is warranted. More specifically, there is a need to prioritize IR testable hypotheses regarding the extent to which an IS has demonstrated one or more of the following, relative to an appropriate active-control implementation strategy: superior *effectiveness* (upper left quadrant [ULQ]) and/or *cost-effectiveness* (upper right quadrant [URQ]), non-inferior *effectiveness* (lower left quadrant [LLQ]) and/or *cost-effectiveness* (lower right quadrant [LRQ]). Each priority testable hypothesis is described below and in Table 4.

**Table 4:** The priority testable hypotheses for implementation research

Priority testable hypothesis	Type
Cost-effectiveness hypotheses from a superiority trial	URQ hypotheses
Effectiveness hypotheses from a superiority trial	ULQ hypotheses
Effectiveness hypotheses from a non-inferiority trial	LLQ hypotheses
Cost-effectiveness hypotheses from A non-inferiority trial	LRQ hypotheses

*URQ* upper right quadrant, *ULQ* upper left quadrant, *LLQ* lower left quadrant, *LRQ* lower right quadrant

#### Effectiveness hypotheses from a superiority trial

Testing the extent to which an experimental IS has superior effectiveness, relative to an active-control IS, is termed IR testing an *upper left quadrant* (ULQ) hypothesis. In contrast to research on clinical treatments, where an active-control condition may not exist or be appropriate, IR should include the most appropriate active-control IS possible. One of the most appropriate active-control condition IS may be the IS used as part of an EBPs effectiveness research. To date, the “large and growing evidence base relating to the effectiveness of implementation strategies” noted by Foy et al. [21] has tested ULQ hypotheses and supports that this testable hypothesis is and should remain a priority for IR. Indeed, given that tests of ULQ hypotheses may continue to be the most common type of IR hypotheses, it may not be much longer before results of ULQ hypothesis tests are analyzed as part of a meta-analysis.

#### Cost-effectiveness hypotheses from a superiority trial

Testing the cost-effectiveness of an IS that has been shown to have superior effectiveness, relative to an active-control IS, is termed IR testing an *upper right quadrant* (URQ) hypothesis. It is considered a priority testable hypothesis for IR as knowing the effectiveness of an intervention/strategy is not sufficient for many potential users, especially decision makers who need to know whether the benefits from the intervention/strategy are commensurate with its costs (i.e., whether it delivers value) [35–38]. Further, noting that economic evaluation of implementation strategies “has been neglected,” Foy et al. encouraged IR with an economic evaluation component [21]. Building upon Garner et al. [31] which found pay-for-performance to be an effective IS for improving the implementation and effectiveness of A-CRA in a superiority trial, Garner et al. [39] provide an example of IR testing an URQ hypothesis. Supporting the cost-effectiveness of a pay-for-performance IS, Garner et al. [39] found that although the pay-for-performance strategy led to 5% higher average total costs compared to the implementation-as-usual control condition, this average cost increase of 5% resulted in a 325% increase in the average number of patients who received target A-CRA (i.e., the EBMI) [39].

#### Effectiveness hypotheses from non-inferiority trial

Testing the extent to which an experimental IS has non-inferior effectiveness, relative to an active control IS, is termed IR testing a *lower left quadrant* (LLQ) hypothesis. Similar to how Schumi and Wittes [40] explain non-inferiority, testing a non-inferiority hypothesis seeks to provide evidence that the IS being tested is “not unacceptably worse” than the IS being used as a control. This is a priority for IR given strategies used to study a clinical intervention’s effectiveness may not be possible in practice settings (e.g., too intensive). We are not aware of IR that has tested LLQ hypotheses. However, a close example is a non-randomized observational IR study by Stirman et al. [41] that compared two strategies for providing post-workshop consultation in an evidence-based cognitive therapy. As detailed by Stirman et al., results of their study did not support the hypothesis of the group consultation and feedback condition being non-inferior to the gold-standard individual feedback condition [41].

#### Cost-effectiveness hypotheses from non-inferiority trial

Testing the cost-effectiveness of an IS shown to have non-inferior effectiveness, relative to an active-control IS, is termed IR testing a *lower right quadrant* (LLQ) hypothesis. Again, given decision makers’ desire to know the extent to which benefits from an IS are commensurate with its costs [35], LLQ hypotheses were identified as a priority for IR. Although not from the field of IR, an example of testing cost-effectiveness hypotheses from a non-inferiority trial is provided by Bansback et al. [42] which extended research by Oviedo-Jockes et al. [43] to support the non-inferiority of injectable hydromorphone hydrochloride (i.e., a narcotic pain reliever) relative to injectable diacetylmorphine hydrochloride (i.e., pharmaceutical heroin).

### Objectives

The primary objective of the PATH4IR Project’s scoping review is to advance understanding regarding the extent to which IR to date has examined the four priority domains, three priority aims, and four priority testable hypotheses described above. We hypothesize that IR addressing these priorities will be limited (i.e., represent significant gaps in the extant IR literature). Thus, a secondary objective of this review is to help advance understanding regarding what domains, aims, and testable hypotheses IR has focused on to date.

## Methods

The scoping review approach developed by Arksey and O’Malley [44] and advanced by Levac et al. [45] guided this scoping review protocol and is therefore organized around five stages: (1) identifying the research question, (2) identifying relevant studies, (3) selecting studies, (4) charting the data, and (5) collating, summarizing, and reporting results. Each stage is described below.

### Stage 1: identifying the research questions

The primary research questions our research team will answer with this scoping review is to what extent have the four priority domains, three primary aims, and four priority testable hypotheses described above been addressed by IR to date? Via an iterative process, our research team also identified the following secondary research questions: (1) which other domains have been studied by IR to date, (2) which other aims have been studied by IR to date, and (3) which other hypotheses have been examined by IR to date.

### Stage 2: identifying relevant studies

*Implementation Science* has been the leading journal for publishing IR (receiving over 800 submissions annually) since its inception in 2006 [46]. In recognition of the field’s rapid growth in the last several years, two additional journals focusing on IR were launched: *Implementation Science Communications* and *Implementation Research and Practice*. This review will focus on IR published in these three journals since their inception. To identify relevant studies, we will search PubMed using the search strategy below and cross-reference the results with lists of publications on the journals’ websites:

Search “Implementation science IS”[Journal] OR (“Implementation science communications”[Journal])) OR (“Implementation research and practice”[Journal])

Filters: publication date to December 31, 2020

Primary research articles published through 2020 are eligible. Articles labeled as “systematic review,” “methodology,” “conceptual paper,” “debate,” “viewpoint,” “commentary,” “letter to the editor,” “practical implementation report,” or “conference proceedings” are not eligible as this review aims to map original IR. Study protocols are also excluded given that intended analyses do not always align with published results. Research articles and short reports will be excluded if the review team agrees that the paper’s primary objective is more aligned with an excluded article type.

### Stage 3: study selection

Reference information and full texts for all articles published in *Implementation Science*, *Implementation Science Communications*, and *Implementation Research and Practice* in 2020 or earlier will be imported into an EndNote database. The articles will be sorted by a reviewer by type to identify all articles labeled by the journal as research articles or short reports. In the subsequent stages, if a reviewer encounters an article deemed ineligible (i.e., labeled by the journal as a research article or short report but is not considered primary IR), the reviewer will raise it with the review team so that consensus around an inclusion decision can be reached.

### Stage 4: charting the data

Table 5 provides a list of variables to be included in the project’s data-charting form, which was developed based on discussions by the review team regarding what information should be recorded for each eligible article and a pilot test of the form with five articles. First author, title, publication year, and article type are included as article identifiers. We will extract whether and which IS, EBMI, HHRO, or CRMM was studied, which relationships between these domains were studied (i.e., path C, A, or B), and whether URQ, ULQ, LLQ, or LRQ hypotheses were tested when studying these relationships to answer our primary question of the extent to which the priority domains, aims, and testable hypotheses have been assessed in IR to date. As a secondary question, we will seek to understand what other domains, aims, and testable hypotheses have been examined by IR to date. For example, we will extract whether studies consider implementation outcomes that are not evidence-based or contextual factors not as moderators or mediators to understand which other domains have been examined and the extent to which they have been examined. We anticipate identifying IR that focused on implementation outcomes rather than EBMIs and therefore will record whether the IS ➔ implementation outcome relationship was assessed. Our form also will include a space to capture other aims and testable hypotheses that IR has examined to date.

**Table 5:** Data elements

Variable	Format	Description
Article identifiers		
First author	Free text	Last name of the article’s first author
Title	Free text	Title of the article
Publication year	Numerical	Year in which the article was published
Article type	Categorical	Whether the article is labeled as a research article or short report by the journal
Primary question: to what extent have the PATH4IR Project’s priority domains, aims, and testable hypotheses been studied in IR to date?		
IS	Dichotomous	Whether the study develops or assesses an IS
	Categorical	If yes, whether the implementation strategies of interest are evaluative and iterative, provide interactive assistance, adapt and tailor to context, develop stakeholder interrelationships, train and educate stakeholders, support clinicians, engage consumers, utilize financial strategies, or change infrastructure
	Free text	If yes, lists the IS of interest
HHRO	Dichotomous	Whether the study assesses an HHRO
	Free text	If yes, lists the HHRO of interest
EBMI	Dichotomous	Whether the study assesses an EBMI
	Free text	If yes, lists the EBMI of interest
CRMM	Dichotomous	Whether the study assesses a contextual factor as a moderator or mediator in some relationship
	Categorical	If yes, whether the contextual factors of interest are related to intervention characteristics (e.g., complexity), outer setting (e.g., external policies and incentives), inner setting (e.g., leadership engagement), individual characteristics (e.g., staff perceptions about the intervention), or the implementation process (e.g., extent of planning ahead of implementation)
Path C	Dichotomous	Whether the study assessed the IS ➔ HHRO relationship
Path A	Dichotomous	Whether the study assessed the IS ➔ EBMI relationship
Path B	Dichotomous	Whether the study assessed the EBMI ➔ HHRO relationship
URQ hypothesis	Dichotomous	Whether the study tested a URQ hypothesis
ULQ hypothesis	Dichotomous	Whether the study tested a ULQ hypothesis
LLQ hypothesis	Dichotomous	Whether the study tested an LLQ hypothesis
LRQ hypothesis	Dichotomous	Whether the study tested an LRQ hypothesis
Secondary question: Which other domains have been studied in IR to date?		
Implementation outcome	Dichotomous	Whether the study assesses an implementation outcome that is not yet evidence-based
	Categorical	If yes, whether the implementation outcomes of interest are related to acceptability, adoption, appropriateness, feasibility, fidelity, implementation cost, penetration, or sustainability
	Free text	If yes, lists the contextual factors of interest
Context generally	Dichotomous	Whether the study considers the implementation context without assessing it as a moderator or mediator in some relationship
	Categorical	If yes, whether the contextual factors of interest are related to intervention characteristics (e.g., complexity), outer setting (e.g., external policies and incentives), inner setting (e.g., leadership engagement), individual characteristics (e.g., staff perceptions about the intervention), or the implementation process (e.g., extent of planning ahead of implementation)
	Free text	If yes, lists the contextual factors of interest
Other domain	Free text	Lists domains other than IS, HHRO, EBMI, implementation outcomes, CRMM, or context generally that are studied
Secondary question: Which other aims have been studied in IR to date?		
Path A-ish	Dichotomous	Whether the study assessed the IS ➔ implementation outcome relationship
Other aim	Free text	Lists relationships other than path C, path A, path A-ish, and path B that are studied
Secondary question: Which other hypotheses have been tested in IR to date?		
Other hypothesis	Free text	Lists testable hypotheses other than URQ, ULQ, LLQ, and LRQ that are studied

*URQ* upper right quadrant, *ULQ* upper left quadrant, *LLQ* lower left quadrant, *LRQ* lower right quadrant

To ensure validity of the form, data will be extracted by a primary reviewer and confirmed by a secondary reviewer for approximately one-third of the included articles. Any conflicts will be discussed until consensus is reached. Clarifications and additional revisions to the data-charting form based on the types of conflicts that arise will be considered. Once the form is finalized at this stage, data from the remaining articles will be extracted by a single reviewer.

### Stage 5: collating, summarizing, and reporting the results

**Fig. 3:**
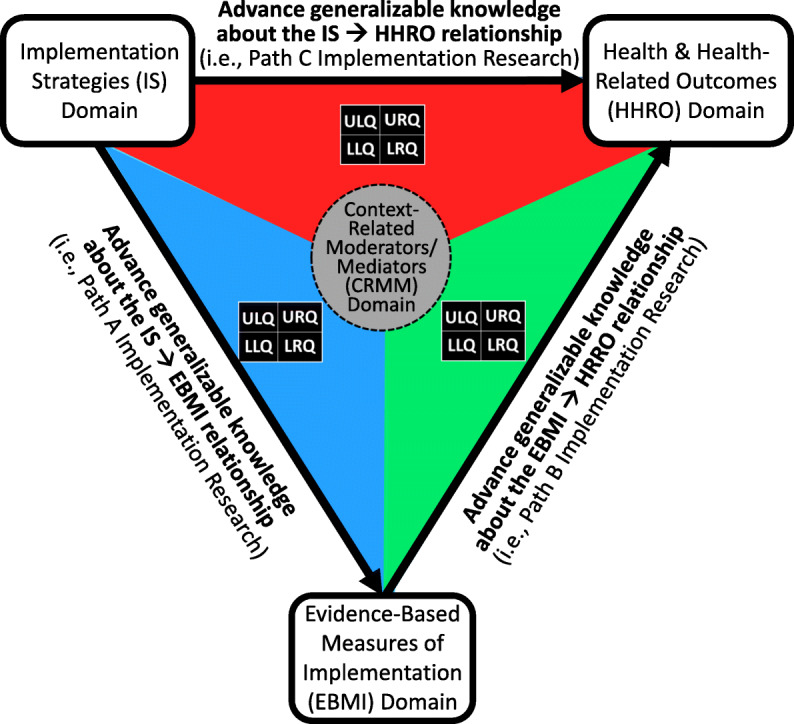
The PATH diagram for implementation research

A PRISMA flow diagram will be used to report results of the scoping review. Additionally, we will present a descriptive overview (including tabular and/or graphical summaries) of the eligible full texts. Because scoping reviews seek to provide an overview of the identified evidence base rather than synthesize findings from across studies, we plan to use our data-charting form to provide a descriptive overview of IR to-date and summarize the research via one or more summary tables (e.g., for each priority aim). Additionally, we will use the PATH diagram (see Fig. 3), which integrates the four priority domains, three priority aims, and four priority testable hypotheses, to develop a map of the evidence.

## Discussion

Despite significant amounts of funding, time, and effort, the field of IR has yet to develop scientific theories as defined by the National Academy of Sciences (i.e., a comprehensive explanation of some aspect of nature that is supported by a vast body of evidence). The findings from this project are intended to help accelerate IR focused on one or more of the identified IR priority aims and testable hypotheses, which in turn will accelerate IR’s development of National Academy of Sciences-defined scientific theories and, subsequently, improvements in public health. Our review is restricted to English-language articles published in three implementation-focused journals, which is a limitation given that IR can be submitted and published elsewhere and in other languages. However, limiting our review to primary research published in these journals provides an efficient starting place given the research has already been screened and deemed relevant to IR. Further, we believe this approach is justified given the purpose of this scoping review is to obtain an initial sense of the IR literature base. Results of this scoping review will be disseminated via presentations at professional conferences (e.g., Annual Conference on the Science of Dissemination and Implementation in Health, Society on Implementation Research Collaboration), publication in a peer-reviewed journal (e.g., *Implementation Science*, *Implementation Research and Practice, Implementation Science Communications*).

## Data Availability

Not applicable.

## Abbreviations

A-CRA: Adolescent community reinforcement approach
CRMM: Context-related moderators/mediators
EBMI: Evidence-based measures of implementation
EBP: Evidence-based practice
HHRO: Health and health-related outcomes
IR: Implementation research
IS: Implementation strategy
LLQ: Lower left quadrant
LRQ: Lower right quadrant
PATH: Priority aims and testable hypotheses
ULQ: Upper left quadrant
URQ: Upper right quadrant
